# Chloroplast Glutamine Synthetase, the Key Regulator of Nitrogen Metabolism in Wheat, Performs Its Role by Fine Regulation of Enzyme Activity via Negative Cooperativity of Its Subunits

**DOI:** 10.3389/fpls.2018.00191

**Published:** 2018-02-19

**Authors:** Edit Németh, Zoltán Nagy, Attila Pécsváradi

**Affiliations:** ^1^Department of Plant Biology, University of Szeged, Szeged, Hungary; ^2^Doctoral School in Biology, Faculty of Science and Informatics, University of Szeged, Szeged, Hungary; ^3^Centre for Agricultural Research, Hungarian Academy of Sciences, Martonvásár, Hungary; ^4^Cereal Research Non-profit Ltd., Szeged, Hungary

**Keywords:** allosteric behavior, glutamine synthetase, negative cooperativity, nitrogen metabolism, *Triticum aestivum*

## Abstract

Glutamine synthetase (GS) is of central interest as the main route of ammonia assimilation in plants, and as a connection point between the organic and inorganic worlds. Even though GS activity is critical for producing high yields of crop plants, the autoregulation of substrate consumption of wheat GS remained unknown until now. Here we show kinetic evidence, that the chloroplast localized GS isoform (GS2) of wheat (*Triticum aestivum* L. cv. Jubilejnaja-50) takes place at the carbon-nitrogen metabolic branch point, where it is a mediator, and its enzymatic activity is regulated in a negatively cooperative allosteric manner. We have discovered that GS2 activity is described by a tetraphasic kinetic curve in response to increasing levels of glutamate supply. We constructed a model that explains the kinetic properties of glutamate consumption and this unique allosteric behavior. We also studied the subunit composition of both wheat leaf GS isoenzymes by a combination of two dimensional gel electrophoresis and protein blotting. Both leaf isozymes have homogeneous subunit composition. Glutamate is both a substrate, and an allosteric regulator of the biosynthetic reaction. We have concluded on the basis of our results and previous reports, that wheat GS2 is probably a homooctamer, and that it processes its substrate in a well-regulated, concentration dependent way, as a result of its negatively cooperative, allosteric activity. Thus, GS2 has a central role as a regulator between the nitrogen and the carbon cycles via maintaining glutamine-glutamate pool in the chloroplast on the level of substrates, in addition to its function in ammonia assimilation.

## Introduction

Metabolism is based on finely regulated, interconnected systems, operated by enzymes that are responding to multiple inputs. The details of enzyme fine tuning are gradually being discovered and described. The emerging picture is complex, revealing multitasking intermediates that are substrates, products and regulatory elements, or signaling molecules at the same time (Floková et al., 2016). The intracellular level of such key molecules must be carefully controlled for mediating adequate cellular and physiological responses. Consequently at critical steps of cyclical processes or at branch points connecting pathways, key enzymes, act as finely tuned switches, allowing homeostasis in rapidly changing biochemical environments (Bush et al., 2012). These enzymes typically exhibit allosteric behavior.

Allosteric effects characterize ligand receptor interactions (Harman, 2001), enzyme regulation (Harlow and Halpert, 1998; Kurganov, 2000; Hegazy et al., 2013) and binding protein–ligand interactions. Best researched examples include the allosteric regulation of hemoglobin (Hill, 1910; Epstein, 1971; Brittain, 1991). Regulatory ligands (or substrates) bind to their target proteins and bring about intramolecular and intermolecular changes resulting, in changes of affinity for the substrate and corresponding changes in enzymatic reaction rates.

Allosteric cooperativity in multi subunit complexes can be both positive and negative (Koshland, 1996). Several mathematical models were constructed to describe allosteric cooperativity Monod-Wyman-Changeux (Monod et al., 1965) and Koshland-Némethy-Filmer models (Koshland et al., 1966; Levitzki and Koshland, 1969). Enzymes exhibiting allosteric cooperativity occupy key positions in interconnected metabolic networks (LaPorte et al., 1984; Kurganov, 2000; Bush et al., 2012).

Glutamine synthetase (GS, EC 6.3.1.2) is one of the pivotal enzymes in metabolism. Even though sessile and motile life forms developed different strategies for obtaining nitrogen supply and its utilization, it is widely accepted that GS has a key role in nitrogen metabolism in all organisms studied so far (Bao et al., 2015; Hakvoort et al., 2017). Numerous isoforms have been identified from a wide range of species with highly conservative sequences (Pushkin et al., 1985; van Rooyen et al., 2011). Eukaryotic GS catalyzes the formation of glutamine from glutamate and ammonia with the concomitant hydrolysis of ATP into ADP and P_i_ (Eisenberg et al., 2000).

In plants GS functions as the key assimilatory enzyme for ammonia, whether derived from N_2_ fixation, nitrate, ammonia nutrition, photorespiration, or the breakdown of proteins and nitrogen transport compounds. Even though there are other ammonia consuming enzymes in plants, GS is the first and only one that is able to bind the inorganic ammonium ion to an organic compound in the concatenation of the enzymes of the nitrogen uptake pathway (Miflin and Habash, 2002). Hence, GS is essential for normal plant growth and development (Bao et al., 2015).

Glutamine and glutamate are pivotal amino acids in plant metabolism. Glutamine, the product of GS, is one of the most abundantly transported amino acids in phloem sap (Caputo et al., 2009) and it serves as starting material for biosynthesis of other amino acids, chlorophylls, tricarboxylic acid cycle intermediates and its amide group serves as nitrogen source for transamination the biosynthesis of numerous metabolic intermediates (Lea and Miflin, 2003). The consumed substrate glutamate is also a reaction partner in multiple pathways (including that of chlorophyll biosynthesis), and is also transported via the phloem. Under different nitrogen supply regimes the glutamine/glutamate ratio of phloem sap was reported to be affected. That ratio may act as a long distance signal (Caputo et al., 2009). Moreover, plant glutamate receptors have been discovered (Tapken et al., 2013) and became the center of interest (Vatsa et al., 2011; Cheng et al., 2016), but the correlation of these observations was not investigated.

Two GS isoforms from wheat (*Triticum aestivum*) leaves: the cytoplasmic GS1 and the chloroplast GS2 are separable by native polyacrylamide gel electrophoresis (PAGE) (Pécsváradi et al., 2009). These distinct isoforms of GS are encoded by a small family of nuclear genes, whose expression is differentially regulated by light and nitrogen supply in a developmental stage and tissue-specific manner (Larios et al., 2004). The activity of plant GS protein can be altered by the binding of 14-3-3 proteins (Finnemann and Schjoerring, 2000), redox-state (Choi et al., 1999), or even the presence of nitrogen monoxide (Melo et al., 2011).

GS1 isoform has a prominent role in the determination of the amino acid composition of the phloem sap, since it is the dominant isoform in phloem companion cells (Caputo et al., 2009). GS1 is particularly important for the process of ammonia assimilation from various sources, such as primary nitrogen assimilation and nitrogen remobilization during different developmental stages or leaf senescence, when nitrogen is remobilized to supply the reproductive sinks (Bernard et al., 2008). Previously we reported that GS1 of leaves gains added significance during the sequential senescence of wheat caused by drought stress, and the changes of GS1/GS2 ratio (Nagy et al., 2013).

One major role of GS2, the predominant isoform in C3 plants, is in the re-assimilation of photorespiratory ammonium (Igamberdiev et al., 2014). The other important function of GS2 is the formation of glutamine in the chloroplast, from where it can be transported from “source to sink” or can be exchanged for various biochemical products via carboxylate transporters of the chloroplast (Flügge, 1998; Weber and Fischer, 2007). The glutamine produced can also participate in the metabolic processes mentioned above. Thus, the activity of GS2 affects the formation of other products such as proline, aspartate, or serine (Brugière et al., 1999).

Some three dimensional models were constructed for the distinct isoforms, presenting different symmetric properties of various oligomers (Pushkin et al., 1985; Torreira et al., 2014). The well-known prokaryotic dodecamer (van Rooyen et al., 2011) and the eukaryotic decamer of *Zea mays* (Yamashita et al., 1989) both consist of dimers arranged into a double ring shape, where the interacting surface of monomer-pairs gives the symmetry plane between the two rings. Structural studies of the octameric GS of *Phaseolus vulgaris* showed different assemblage of subunits, where one ring is built by two dimers lying next to each other creating a tetramer and another similar tetramer ring positioned underneath and the two rings rotated 90° relative to each other. The complete octamer is presumed to have four catalytically active sites and eight predicted ATP binding surfaces (Llorca et al., 2006). However, no kinetic evidence has been provided to prove this model in other plants and even the half-site reactivity of GS remained a presumption until now in plants.

A few reports dealt with the allostery of eukaryotic GS, however no physiological interpretation, supported by detailed biochemical analysis was proposed in either one (Guiz et al., 1979; Mann et al., 1980; Ahmad et al., 1982). Neither the results of high resolution kinetic measurements of substrate utilization, nor apparent data about the subunit composition of the wheat GS2 holoenzyme have been available previously. Furthermore, supporting theories between published kinetic characteristics and structural properties of plant GSs are still barely present in the literature.

On one hand, in its physiological role GS functions as the major ammonia assimilatory enzyme. But on the other hand, GS is also a carbon skeleton consumer and it is important to remember that, in plants, carbon utilization is energetically expensive. Moreover, the known transcriptional and post transcriptional regulatory mechanisms (Deuel and Prusiner, 1974; Nkoa et al., 2003) are not necessarily the fastest ways to produce an immediate change to control the glutamate-glutamine ratio which suggests the existence of fine regulation.

We conclude that GS2 plays a role in balancing nitrogen and carbon metabolisms in wheat leaves through its special allosteric regulation. We show that glutamate acts as a regulator substrate of the biochemical reaction, and characterize the allosteric behavior of GS. We present kinetic data, to support the suggested subunit composition and the fine regulation of GS, showing its strategical importance in plant homeostasis.

## Materials and methods

### Plant material

Seeds of wheat (*T. aestivum* L. cv. *Jubilejnaja-50*) were first soaked in tap water for 2 h and subsequently germinated for 1 day at 24°C on wet filter paper in Petri dishes in a germination chamber. Seedlings were transferred to 0.5 mM CaSO_4_ for 2 days in a growth room with a photoperiod 12 : 12 h, light: dark, with 200 μmol m^−2^ s^−1^ photon flux density at temperature of 25°C. Subsequently, the CaSO_4_ solution was exchanged for a modified Hoagland solution (Zsoldos et al., 1986).

### Protein extraction

Crude extracts for experimental procedures were produced from first (oldest) leaves of 1 week old seedlings. Samples were collected and used immediately. Leaves were homogenized in a mortar with 1:3 (w:v) ratio with extraction buffer−200 mM Tris-HCl, 1 mM reduced L-glutathione, 10% glycerol, 10 μl ml^−1^ protease inhibitor cocktail (Sigma Aldrich, St Louis), pH 7.5. Samples were centrifuged at 4°C, 10 min, 13,000 g. Supernatant was collected and kept on ice until used. The protein content of samples was determined by the Bradford method (Bradford, 1976).

### Identification of glutamine synthetase subunits

Subunits of GS1 and GS2 isoenzymes were identified by immunoblotting after two dimensional separation of the crude extract. The first dimension was run as non-denaturing PAGE (Laemmli, 1970). A lane of the first dimension was cut out and washed [0.5 M Tris-HCl pH 6.5, 10 v/v % glycerol, 32.5 mM dithiothreitol, 2 w/v % sodium dodecyl sulfate (SDS), 2% ß-mercaptoethanol, 0.02% Servablue-G] twice for 1 h. As a second dimension SDS-PAGE was applied. Separating gel contained 10% (w/v), and the stacking gel contained 4% acrylamide (acrylamide:bisacrylamide = 19:1). Protein blot analysis was performed as previously described (Nagy et al., 2013).

### Native enzyme purification

GS2 isoform was separated from crude protein extracts (described above) by native PAGE (Laemmli, 1970), using Mini protean II (BioRad) chambers. Localization of GS2 in identical gels was based on enzyme activity (Pécsváradi et al., 2009). After biochemical visualization a scheme had been prepared for the fine coordination of dislocation of GS2 containing gel sphere for further isolation. GS2 was retrieved by the maceration of gel slices in an ice cold mortar with silica sand and protein extraction buffer in proportion to loaded crude extract volume. Importantly, by avoiding application of high salt concentration or harmful detergents in our purification procedure, the native conformation of the enzyme was preserved. After centrifugation at 4°C for10 min, at 13,000 g, the supernatant was collected and used for kinetic measurements immediately.

### Enzymatic assays

Determination of GS enzyme activity was based on an *in vitro* modified synthetase reaction (Rhodes et al., 1975), where the amount of produced γ-glutamyl monohydroxamate (GMH) is detectable by a stop reaction. The total reaction volume consisted of 75 μl plant sample material and 200 μl reagent buffer. Reagent buffer always contained 21 mM magnesium chloride hexahydrate and basically 36 mM imidazole, 15 mM ATP, 20 mM hydroxylamine, 30 mM Na-glutamate, but concentrations varied depending on the actual kinetic assay; ATP: 0–25 mM; Na-glutamate: 0–200 mM; glutamine: 0–57 mM; GMH: 0–80 nM.

### Data analysis

For non-liner regression analysis of data we used SigmaPlot® (Systat Software). The kinetic models were compared by their corrected Akaike information criteria value (AICc). The presented kinetic parameters were computed by the program. We studied the V_max_ (maximum velocitiy), K_s_ (the substrate concentration belongs to ½ V_max_) and also *n* (Hill coefficient) and K_i_ (concentration of inhibitor, which causes 50% inhibition) as required. Measurements were repeated at least 3 times and the representative data are shown in the article.

## Results

### Identification of subunits

**Figure 1:**
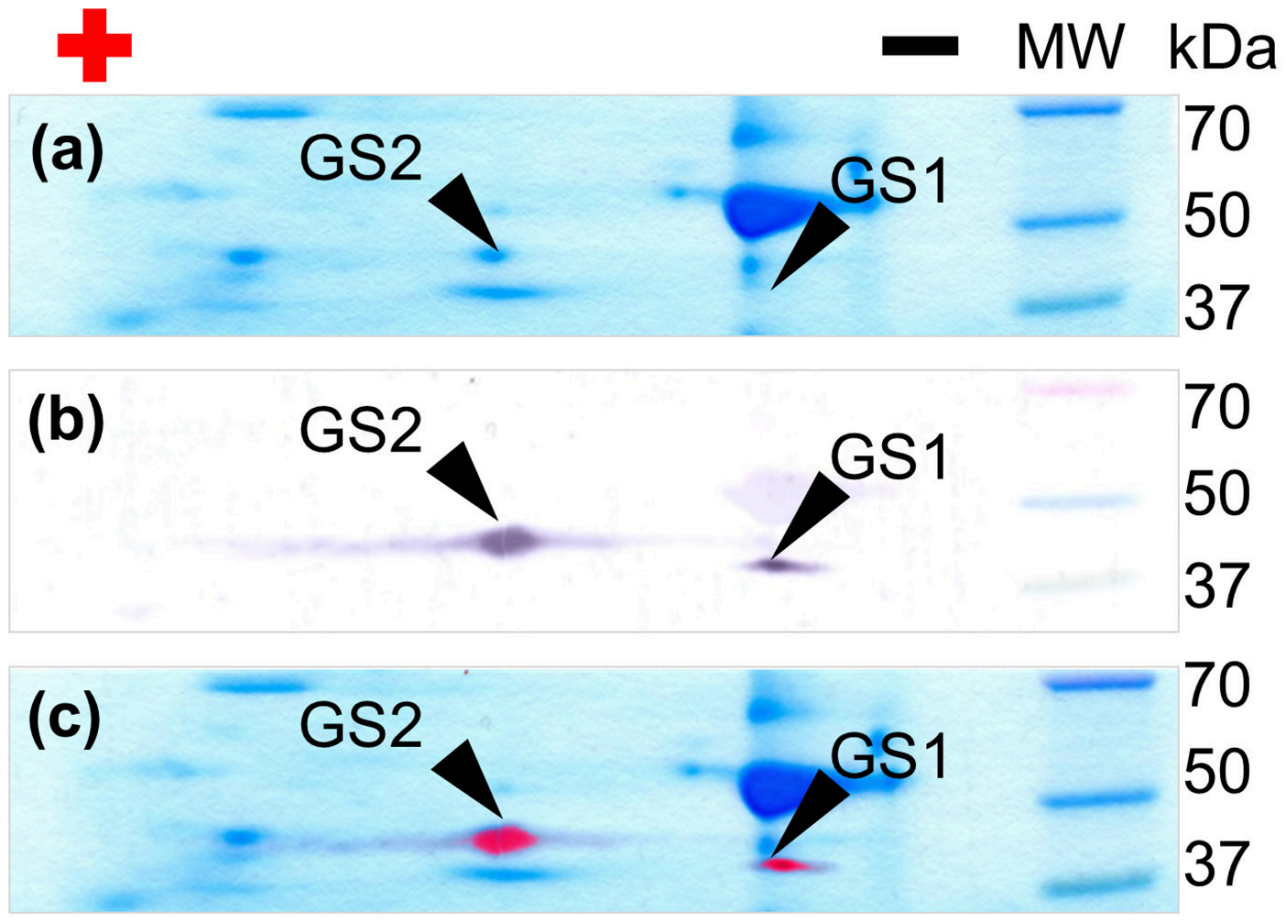
Identification of GS subunits, and study of subunit composition. **(a)** Coomassie Brilliant Blue G stained 2D PAGE separation of crude leaf extract; **(b)** Immunoblot of glutamine synthetase after 2D PAGE separation. **(c)** Merged picture of **(a,b)** showing the different localization of GS isoenzymes in polyacrylamide gel. Plus and minus labels the anode and cathode site of the first dimension respectively. MW: molecular weight standard.

Previously it has been shown by native gel electrophoresis that two isoforms of GS are present in wheat leaves: the cytoplasmic GS1 and the plastidic GS2 (Pécsváradi et al., 2009). Before the kinetic analysis we studied the subunit composition of wheat leaf GS isozymes. Western blot analysis of two dimensional separation of wheat leaf soluble protein extract resulted in two identical marks on the blot membrane with the calculated molecular weight of 41.7 and 46.4 kDa for GS1 and GS2 subunits respectively (Figure 1, Supplementary Figure 1). Since we did not detected extra shifted bands, we have concluded that both the cytoplasmic GS1 and the plastidic GS2 isoenzyme are homomers.

### Kinetic measurements

These experiments were performed for finding a regulatory substrate, mostly via enzyme activity studies. At least 80% of total GS activity derives from GS2 isozyme (McNally et al., 1983), thus crude extracts are suitable for exploratory kinetic studies.

#### Effect of end-products

##### Gamma-glutamyl monohydroxamate (GMH)

**Figure 2:**
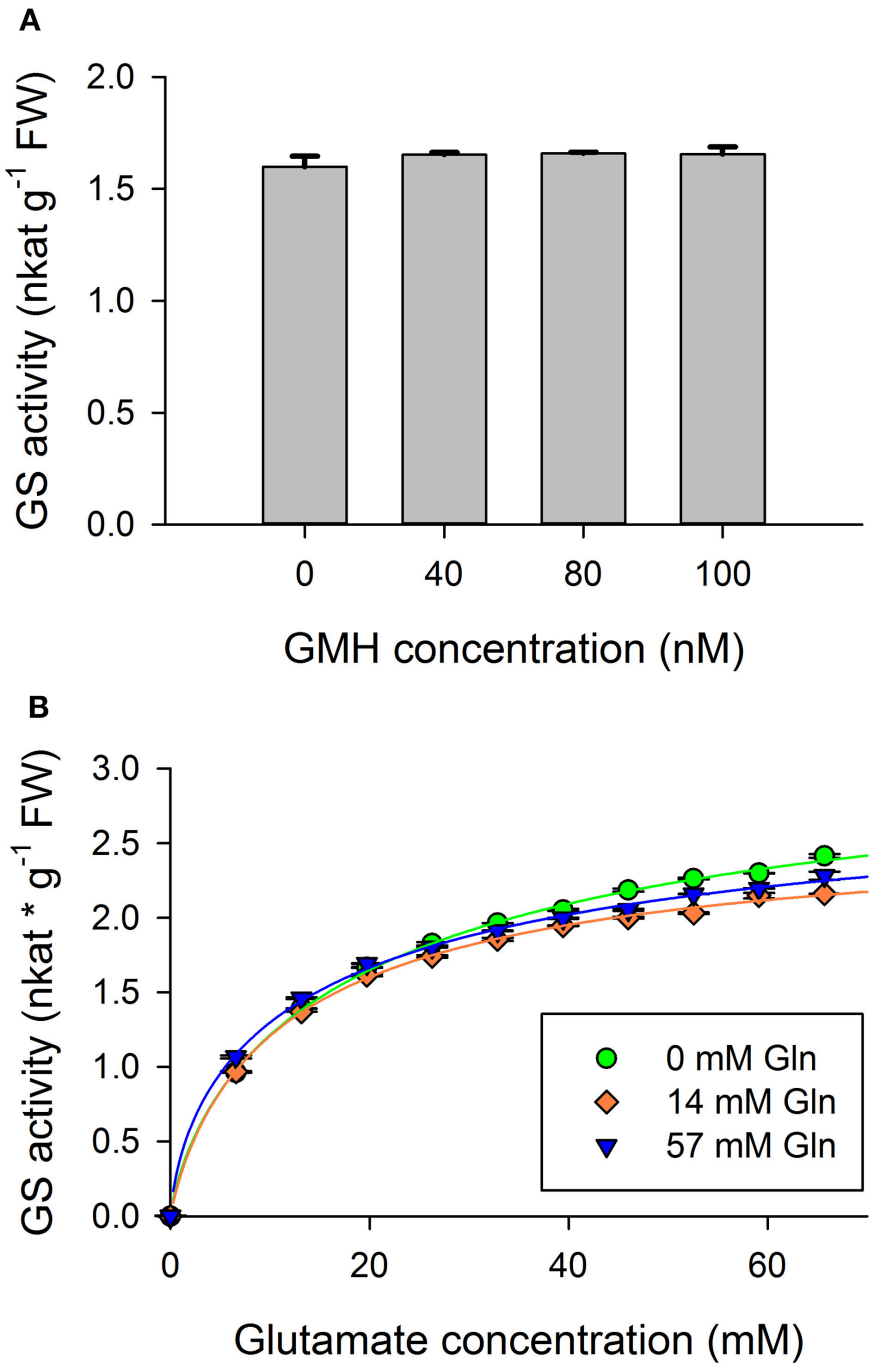
Lack of product inhibition. **(A)** Total glutamine synthetase activity of wheat (*Triticum aestivum* L. cv. Jubilejnaja-50) leaf crude extract *in vitro* with additional gamma-glutamyl-monohydroxamate (GMH), the artefactual product. No significant differences were detectable. **(B)** Total *in vitro* glutamine synthetase (GS) activity in the presence of 0, 14, and 57 mM of additional glutamate, the natural product. There is no correlation between glutamine content and maximal velocity.

Since the modified synthetase reaction produces GMH, it was required to examine whether the GMH itself caused any disturbance in the operation of the enzyme during the experiments. Total GS activity was studied with various GMH concentrations (Figure 2A). No significant changes were detectable under the applied conditions. Thus, the possibility that GMH has any effect on GS activity in our experiments can be excluded.

##### Glutamine

The effect of numerous concentrations of glutamine on total GS activity of wheat leaf protein crude extract was studied (Figure 2B). Curves showed smooth saturation for glutamate supply in case of the applied *in vitro* treatments. Maximum velocity of the curves slightly differs, but this change is not in correlation with glutamine supply conditions. Additional glutamine supplies the glutamine consuming enzymes like GOGAT (glutamine oxoglutarate aminotransferase) or asparagine synthase present in the crude extract (Miflin and Habash, 2002). This can lead to extra glutamate supply during the reaction, which causes the observed early saturation. The resulting data suggest that glutamine does not exert product inhibition in the course of the GS catalyzed enzymatic reaction.

#### Effect of hydroxylamine

**Figure 3:**
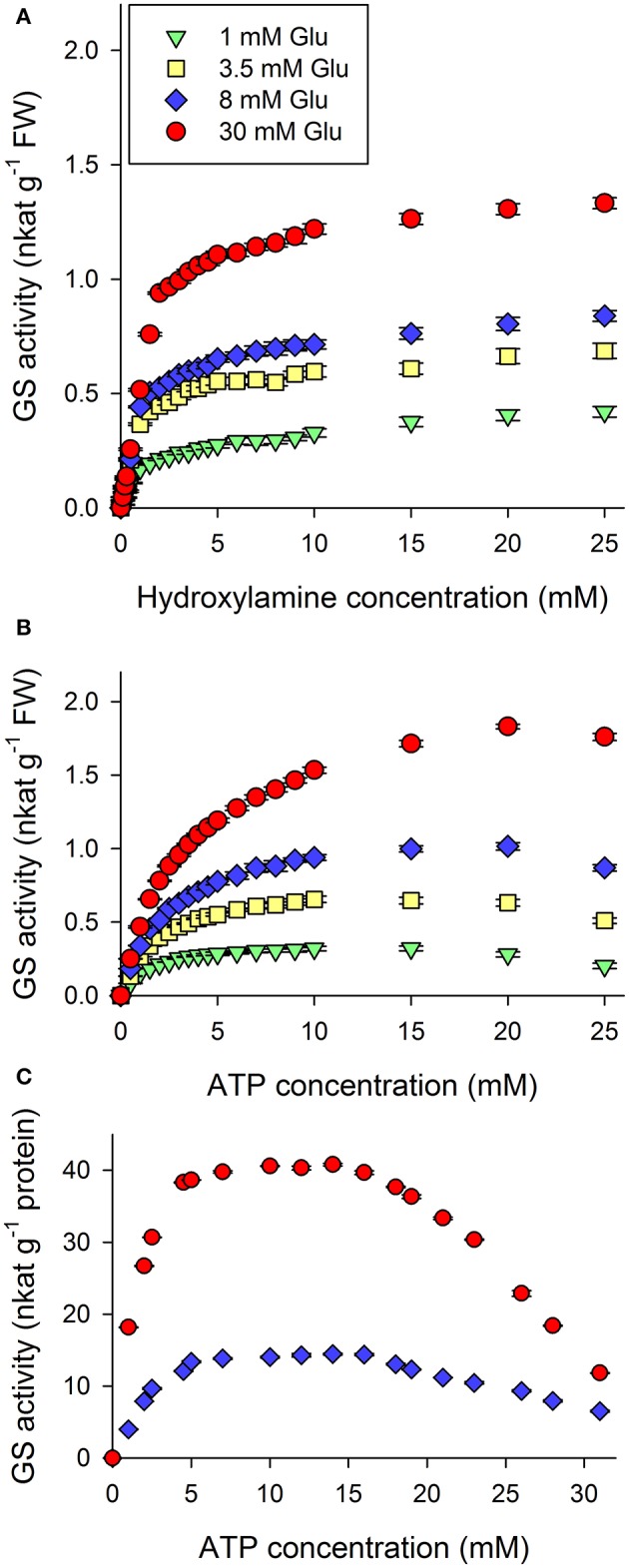
Total *in vitro* glutamine synthetase (GS) activity of wheat leaves crude extract in relation to additional hydroxylamine **(A)** and ATP **(B)** in the presence of 1, 3.5, 8, 30 mM glutamate. ATP saturation of purified GS2 extract **(C)**. Elevated glutamate concentrations increased V_max_ values of both kinetic curves. K_s_ values slightly increase in correlation to glutamate. Hydroxylamine saturation **(A)** occurs at very low concentration. High concentrations of ATP **(B)** caused the inhibition of GS activity. ATP reduces the activity of purified GS2 above 15 mM.

We studied the effect of hydroxylamine; results are shown (Figure 3A). Hydroxylamine was used as the ammonium donor for the experiments. The total GS activity of wheat leaf protein extract was measured in a range of hydroxylamine concentrations. The resulting kinetic curve starts very steeply, and reaches a steady-state phase at a low hydroxylamine concentration. The curve shows a saturation which is ascribable to the lack of an allosteric effect. The observed K_s_ is less than 1.5 mM (Table 1A), which is a physiologically low value. This result indicates the high hydroxylamine affinity of GS.

**Table 1:** Kinetic parameters of curves shown on Figures 3A,B.

**(A)**			
**Glutamate (mM)**	**V_max_ (nkat g^−1^ FW)**	**K_s_ (mM)**	
1	46.82 ± 1.73	2.00 ± 0.27	
3.5	77.07 ± 1.39	1.07 ± 0.09	
8	95.09 ± 1.89	1.23 ± 0.11	
30	162.73 ± 3.76	1.45 ± 0.14	
**(B)**			
**Glutamate (mM)**	**V_max_ (nkat g^−1^ FW)**	**K_s_ (mM)**	**K_i_ (mM)**
1	0.5 ± 0.05	2.64 ± 0.53	26.02 ± 6.85
3.5	0.98 ± 0.07	3.04 ± 0.41	39.80 ± 8.74
8	1.34 ± 0.08	3.19 ± 0.39	83.11 ± 25.88
30	2.06 ± 0.09	3.49 ± 0.30	450500 ± 4.*E*+08

***(A)** Parameters of Michaelis-Menten kinetics fitted to hydroxylamine kinetic dataset (Figure 3A). **(B)** Parameters of uncompetitive substrate inhibition kinetic curves fitted to ATP kinetic curves dataset (Figure 3B). Growing K_i_ shows the decrease of the inhibitory effect of ATP in correlation with growing glutamate supply. Values are mean ± 1 SE. V_max_ is maximal velocity and K_s_ is the substrate concentration at ½ the maximum velocity, K_i_ is the ATP concentration decreasing the maximum velocity by 50%*.

#### ATP saturation

High resolution kinetic measurements with ATP (Figure 3B) resulted in a curve, which did not show as high an affinity of the enzyme to ATP as to hydroxylamine. There were no repeatable break points on the saturation kinetic curves. Decreasing enzyme activity has been observed at extremely high ATP concentrations. The physiological value reported to be around 2–3 mM (De Col et al., 2017). In reflection of our results *in vitro* extremely high ATP concentration has a decreasing effect in crude extracts above 20 mM. We also studied ATP saturation in purified extracts (Figure 3C). The curves shifted to the left, indicating an earlier saturation and the observed decrease in activity shifted to 15 mM. The analysis did not show any convincing kinetic evidence for allosterically cooperative effect of ATP below this concentration.

#### Glutamate saturation kinetics

##### Experiments on crude extract

**Figure 4:**
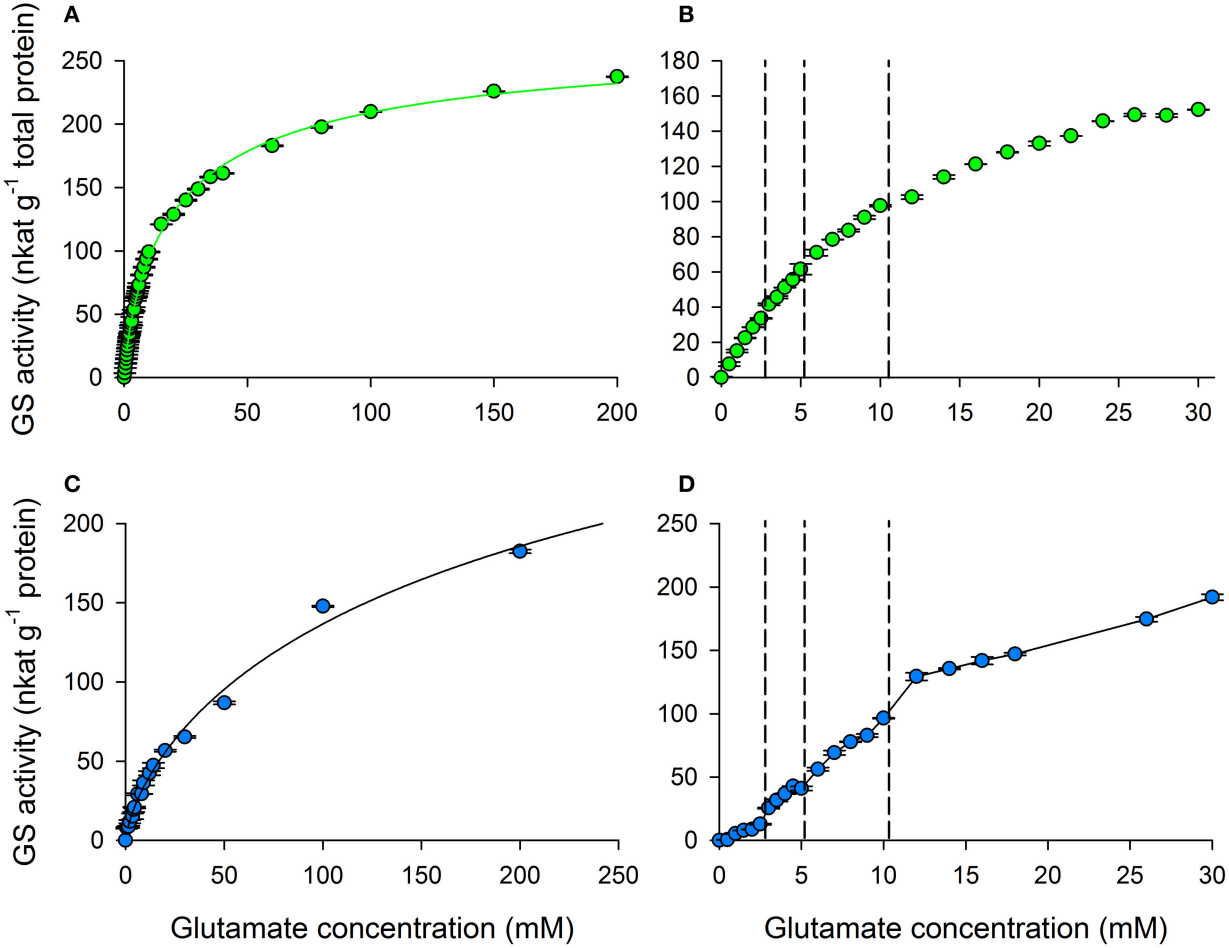
Breakpoints of glutamate kinetic curves. **(A)** Wide interval glutamate kinetics of crude glutamine synthetase (GS) protein extract with the best-fit curve of Hill equation. **(B)** Total activity of crude GS protein extract in a low glutamate concentration interval. **(C)** Activity of purified GS2 with the best-fit curve of Hill equation. **(D)** Activity of purified GS2 protein extract in a low glutamate range. The concentrations which belong to break points (vertical dashed lines) are identical to those observed when using non-purified samples.

Kinetic studies on glutamate dependence of total GS activity were also performed. The effect of glutamate was examined with a wide range of glutamate supply such as 0–200 mM in experiments with crude extracts. The resulting curve showed specific characteristic (Figure 4A). Analyzing the data by SigmaPlot revealed that the curves can be accurately described by the Hill equation, with Hill coefficient *n* = 0.792 ± 0.018, K_s_ = 22.38 ± 1.44 mM and V_max_ = 273.08 ± 5.44 nkat g^−1^ fresh weight. Typically, the activity of enzymes showing negative cooperativity can be described by Hill equation (Hill, 1910; Bush et al., 2012). Important to note here that even if these results are affected by the presence of GS1 in the crude extract, more than 4/5 of the activity originates from GS2.

We examined GS activity (Figure 4B) with a glutamate concentration range, similar to the physiological range. The concentrations of glutamate in the cytosol and the stroma were determined to be 41 and 26.4 mM, respectively (Weber and Flügge, 2002). Our studies revealed a curve with dynamics unique for GS activity. Instead of having a linear slope followed by a steady-state phase a slightly staggered phase has been observed. This staggered curve is divided into four parts by three breakpoints giving the staggered character to it. It can be only observed using accurate, high resolution glutamate concentration scale, without which the details would be lost.

##### Experiments on purified GS2 protein

To provide evidence to show that nothing else but only GS is responsible for this kinetics in the following we used purified enzyme extract. The kinetic parameters were examined with glutamate concentrations ranging from 0 to 200 mM (Figure 4C) to show that the Hill coefficient still indicates the negative cooperativity (*n* < 1). The resulted values were *n* = 0.711 ± 0.056, V_max_ = 425.23 ± 107.59 nkat g^−1^ protein and K_s_ = 285.76 ± 179.47 mM. The corrected Akaike information criteria index was 12 lower (better) for Hill equation against the MM equation. This indicates that there is a greater possibility for GS to exhibit negative allosteric cooperativity than to be non-cooperative. GS activity was examined with an additional glutamate concentration range from 0 to 30 mM (Figure 4D). By the purification we eliminated the interference of GS1 and those biochemical processes which could have provided extra substrate during incubation. As these factors became eliminated, the previously observed staggered character has been shown unequivocally. The steps appear in a glutamate dose-dependent way suggesting glutamate as a regulatory substrate.

#### The effect of glutamate on the saturation of ATP and hydroxylamine

K_s_ values are known to be highly conserved properties of enzymatic reactions. Our hypothesis is that glutamate is a regulator substrate of the enzymatic reaction. Indirect proof of this hypothesis would be the changes of V_max_ and K_s_ values of the kinetic curves for the various concentrations of the hypothetical non-regulator substrates under different glutamate supply conditions. Regulatory effects must result in a change of these kinetic parameters. For these experiments glutamate concentrations between the previously described breakpoints were chosen. We studied the V_max_ and K_s_ parameters (Table 1) of hydroxylamine and ATP kinetic curves under different levels of glutamate supply (Figures 3A,B). These kinetic parameters were elevated with respect to glutamate in every case with an exception of the hydroxylamine curve of 1 mM glutamate supply, which had a K_s_ in between the curves of the 3.5 and 8 mM glutamate supplied reactions. Probably this is a result of a technical artifact resulted by the extremely high affinity of GS for hydroxylamine.

High, 15 mM ATP concentration inhibited the activity of GS. This inhibitory concentration is considered as unnaturally high (Robinson and Portis, 1988; Olsen et al., 1989). The inhibitory concentration required was higher with better glutamate supply. Besides it is clearly shown that the more glutamate is present in the system the higher the glutamine production. Neither ATP nor hydroxylamine was able to limit the catalytic activity of GS, only glutamate itself.

We assume that glutamate plays a regulatory role by modulation of catalytic activity of the subunits via allosteric cooperativity resulting in the characteristic dynamics of the kinetic curve. Based upon these results we created a theoretical model, described below, which explains the operation of the enzyme.

#### Effect of dilution

**Figure 5:**
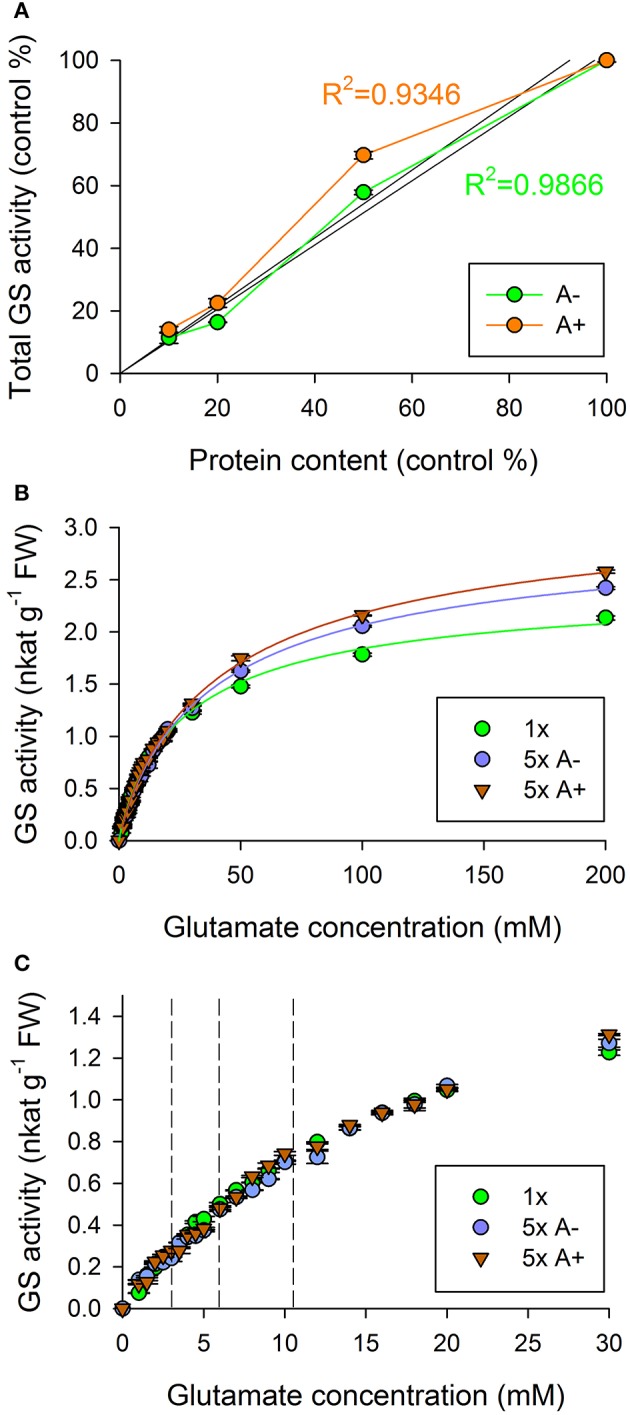
GS enzyme activity of diluted wheat (*Triticum aestivum* L. cv. Jubilejnaja-50) leaf crude extracts. **(A)** Linearity of correlation between the activity and the quantity of the sample material. (Activity values are not corrected by the degree of dilution) Decrease in total GS activity correlates linearly with the amount of sample material without albumin. Activity of albumin treated samples increased, and the correlation is less linear. **(B,C)** Saturation curves of diluted crude extracts. Activity values are corrected by the degree of dilution. A+/A– indicate that the protein content was/ not restored to the original state (1x) with additional albumin. **(B)** The change of maximum velocity in diluted samples, and effect of albumin on saturation. **(C)** Overlapping initial part of saturation curves. Breakpoints of curves range with previously observed glutamate concentrations. Breakpoints indicated by vertical dashed lines.

Assembly of protein complexes is clearly affected by macromolecular crowding. Therefore, we followed the linearity of bovine serum albumin containing and albumin free systems (Figure 5A). We used albumin to compensate for the protein deficit which derives from dilution. Without albumin the system is closely linear, which shows the stability of the enzyme itself. Albumin addition increased the activity of the crude extract, and decreased the linearity of the system. Based on these results, we cannot exclude the presence of an effector or activator in the crude extracts. We also studied the effect of dilution on the kinetic parameters and glutamate dose-response of GS of crude extracts (Figures 5B,C, Table 2). The maximum velocity and the K_s_ increased in case of the diluted samples. However, the glutamate dose-response and the Hill coefficient remained exactly the same in all samples. Thus, the dose-response feature is not affected by macromolecular concentration, but is a result of intramolecular interactions.

**Table 2:** Changes of kinetic parameters as a result of dilution and albumin treatment of crude extract.

**Dilution**	**V_max_ (nkat g^−1^ FW)**	**K_s_ (mM)**	***n***
1x	2.46 ± 0.07	28.97 ± 2.21	0.88 ± 0.02
5x A−	3.01 ± 0.06	41.15 ± 2.43	0.88 ± 0.02
5x A+	3.27 ± 0.10	45.50 ± 3.65	0.87 ± 0.02

*Parameters of Hill equation fitted to dataset shown on Figures 5B,C. Values are mean ± 1 SE. V_max_ is maximal velocity and K_s_ is the substrate concentration at ½ the maximum velocity. A+/A– indicate that the protein content was/ not restored to the original state (1x) with additional albumin*.

### Model description

**Figure 6:**
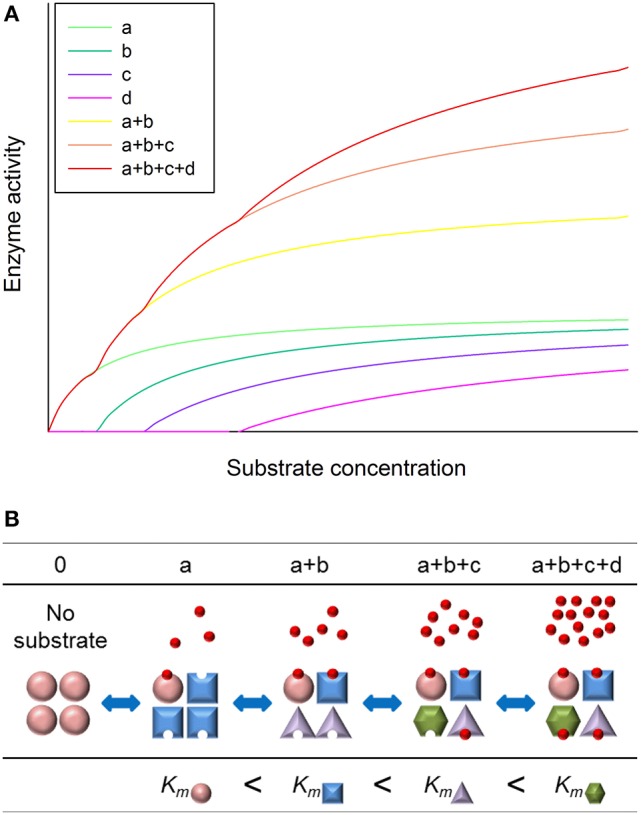
Model of homooctamer GS enzyme operation. **(A)** Plotting of negatively cooperative substrate dependent enzyme operation. Hill equations describe catalytically active units independently: *a*–*d*. Their summations describe the activity of actual states in dependence of glutamate supply. Red curve shows the theoretically observable total GS2 activity. **(B)** Demonstration of activation of GS subunit-pairs. The activation of a subunit-pair indicates the conformational changes of the other unit, while it decreases the substrate affinity. As a result, the different statuses have characteristic K_s_ values.

We created a simplified model to describe the fine regulation of the wheat leaf plastidic glutamine synthetase. The model visualizes enzyme activity for increasing regulator substrate concentration, where GS2 subunits become catalytically active at discrete substrate concentrations. In our model the number of active subunits of the enzyme octamer increases with higher substrate availability (Figures 6A,B).

The measurable enzyme activity is composed of the activity of all the catalytic sites functioning at the time. According to the published structural analysis of GS proteins of a few organisms we assume that a symmetrical dimer of subunits forms an operational unit with one active site. Mathematically the function of one unit can be described by the Hill equation (Hill, 1910).

Based on our observations we assume GS2 to be an octamer, with a possible half-of-the-site reactivity. Hence our model counts with four operational dimers. The sum of velocity of all the subunit pairs gives the actual velocity of the whole octamer:

$$v_{0}=v_{1}+v_{2}+v_{3}+v_{4}$$

Where ν_0_ is the actual velocity of the octamer; ν_1–4_ are actual velocities of subunit pairs. Theoretically it is possible, that the subsequent subunit switches on, when the previous one reached its ½ *V*_*max*_. This means that the total activity can be described by the sum of four Hill equations. Then the equation above can be described, as:

$$\begin{array}{l} v_{0}=\frac{V_{max1}\times {\left[S\right]}^{n}}{K_{s1}^{n}+{\left[S\right]}^{n}}+\frac{V_{max2}\times {\left[S-K_{s1}\right]}^{n}}{K_{s2}^{n}+{\left[S-K_{s1}\right]}^{n}}\\ \;+\frac{V_{max3}\times {\left[S-K_{s2}\right]}^{n}}{K_{s3}^{n}+{\left[S-K_{s2}\right]}^{n}}+\frac{V_{max4}\times {\left[S-K_{s3}\right]}^{n}}{K_{s4}^{n}+{\left[S-K_{s3}\right]}^{n}} \end{array}$$

*V*_*max*1–4_ is maximal velocity and *K*_*s*1–4_ are the substrate concentrations at ½ the maximum velocity of identical subunit pairs; *n* is Hill coefficient, where 0 < *n* < 1. At a very low substrate concentration only one unit works. One initial equation can characterize this state. At the initial interval three of the four equations take on zero value. At a discrete substrate concentration, where an extra unit becomes catalytically active, its activity is added to that of the already active unit. In this simplified model the values taken up by the subsequent functions are equal to the initial one. Thus, when the summation of equations is visualized a break point can be distinguished as the second function increases. Basically what we can observe as the result of glutamate kinetic measurement is the sum of functions systematically shifted to the right. This model shows only the scheme of operation. It is not formulated to deal with the possible transition states, which coexist, in an unknown ratio (Kern and Zuiderweg, 2003).

## Discussion

GS is a connection point of nitrogen and carbon metabolisms, as the first enzymatic component in nitrogen uptake which is able to bind inorganic nitrogen to a carbon skeleton (Torreira et al., 2014). GS is part of the GS-GOGAT-GDH system, which is the major route for ammonium assimilation in plants (Forde and Lea, 2007). Accordingly GS has a prominent role on the map of biochemical pathways. We suggest that GS has a fast response and a very efficient, strict regulation at the level of substrates, which is made possible by allostery.

Previously it has been shown that young wheat leaves contain two isoforms of GS, that are separable by native PAGE: the cytoplasmic GS1 and the plastidic GS2 (Pécsváradi et al., 2009). In our studies the protein blots of the two dimensional PAGE of leaf protein extracts showed, that both isozymes were constructed of separate sets of monomers. Consequently the GS oligomer is a homomeric system, and that observation also applies to wheat GS2. Based on our finding and previous publications (Pécsváradi et al., 2009) GS has the proposed structure of a symmetrical oligomer with more than one binding site.

Following the effects of glutamine and GMH, the natural and the artefactual end-products of the GS catalyzed reaction we showed that GS2 activity is not inhibited by these end-products (Figures 2A,B). However, products of the nitrogen cycle have been recognized as feedback regulators of glutamine production in prokaryotes (Shapiro and Stadtman, 1970; Deuel and Prusiner, 1974).

Our results using hydroxylamine (Figure 3A) indicated an extremely high hydroxylamine affinity and thus also high ammonium ion affinity of GS. Besides the saturation curve did not show any special characteristics under the test conditions, indicating that ammonia does not have any significant regulatory effect on the biochemical reaction.

The solubility of ammonium ion changes drastically with the alteration of pH in aqueous solutions, where lowering pH by one unit results in a tenfold increase of soluble ammonia concentration (Johnson and Berry, 2013). Since there are considerable pH changes in chloroplasts with photosynthetic activity, and ammonia is a cytotoxic compound (Goodall et al., 2013); the high ammonia affinity is one kinetic property which permits GS to play a role as a main ammonia assimilatory enzyme. Besides, previous findings showed that GS has greater affinity for ammonia than glutamate dehydrogenase (GDH) (Nkoa et al., 2003). This also makes GS a main glutamine producer and also the main connection point between nitrogen assimilation and carbon homeostasis.

In our studies high ATP concentrations inhibited glutamine production *in vitro* (Figure 3B). This inhibition could be a result of *in vitro* saturation of ATP binding sites which are not necessary for the stoichiometry of the reaction. ATP caused inhibition is not unprecedented, in the field of binding mechanisms (Olsen et al., 1989). Our result is the biochemical proof of a previous modeling analysis which suggested that a GS dimer has two ATP binding sites (Llorca et al., 2006). Besides we suggest that this inhibitory effect does not have any physiological relevance.

Under different glutamate supply conditions K_s_ parameters of hydroxylamine and ATP slightly shifted in relation to glutamate supply conditions. We suggest that, this shift indicates that glutamate is a possible regulatory substrate.

Studying the kinetic properties of GS2 with a glutamate concentration range of 0–200 mM (Figures 4A,C) we observed that the kinetic curve can be characterized by the Hill equation. This means that the steric tension within the macromolecule is so strong, that the binding of the first substrate impedes the binding of the substrates at the other sites. Furthermore, the lower glutamate concentration range of the curve is staggered, which divides the line into four steps (Figures 4B,D). Therefore, GS2 possibly has a substrate regulated saturation mechanism. This complicated kinetic character of GS2 has not been observed before; such kinetic curve has never been published for plastidic GS at such a high resolution glutamate scale.

We studied the effect of the macromolecular environment by albumin addition and dilution. Albumin can effect on a possible protein-protein interaction in the crude extract by stabilizing, or destabilizing it. We found, that the glutamate dose-response is constant under different conditions. We concluded that this response is due to intramolecular interactions. However, since both K_s_ and V_max_ are slightly affected by dilution, we do not exclude the presence of an effector molecule in the crude extracts. In *Arabidopsis thaliana* an activator protein has been described, that interestingly, does not affect the V_max_ value significantly, but lowers the K_s_ value (Osanai et al., 2017). Thus, the loss of a possible effector like this may lead to the elevation of K_s_. Our diluted sample showed an elevated K_s_ value that can be the result of the dissociation of a potential activator in the crude extract. However, electrophoretic studies of present or previous (Pécsváradi et al., 2009; Nagy et al., 2013) experiments never indicated the presence of a GS2 bound effector.

Importantly, as the glutamate dependence was studied with purified GS2 (Figures 4C,D), the breakpoints of the kinetic curve became more pronounced providing evidence that the staggered kinetic curve is characteristic uniquely of GS2. In previous reports the staggered character of other enzymes is ascribed to saturation of binding sites with different affinity (Levitzki and Koshland, 1969; Traut, 2014).

We suggest that the observed steps are result of different states of the enzyme in response to glutamate supply. Steps start up at discrete glutamate concentrations where the catalytically active units of the enzyme switch on one after another. Our GS2 functional model is an enzyme with four catalytically active states. These states have increasing V_max_ and decreasing affinity respectively with increasing substrate concentration (Figure 4B). Consequently, when only a small amount of carbon skeletons, like glutamate, are available the enzyme has low efficiency, since only one subunit works. However, with increasing amounts of carbon skeletons additional subunits are in operation, increasing the efficiency of the GS2 oligomer. Consequently glutamate takes part in the biochemical reaction not only as a substrate but also as a regulator. As the enzyme shows the characteristics of negatively cooperative allosteric regulation that requires great steric tension, these dimers are likely to have half-of-the-site reactivity as observed in D-galactose specific lectin (Rao and Gowda, 2012).

Due to its special allosteric behavior GS2 is an enzymatic switch and balancer between the carbon and nitrogen metabolisms. Negatively cooperative enzymes have been described at important metabolic branch points previously, such as GDH in Mammalia (Kurganov, 2000) and CTP synthase (Levitzki and Koshland, 1969). The advantage of this type of regulation at critical steps has been discussed (Bush et al., 2012). In case of GS2 this complex allosteric behavior establishes autoregulation at the connection point of nitrogen uptake and carbon skeleton utilization.

Besides all the published GS oligomers are composed of at least eight monomers (Pushkin et al., 1985; Llorca et al., 2006) and we observed four steps of the glutamate kinetic curve. These observations strongly suggest that wheat leaf GS2 is a homooctamer composed of four pairs of monomers, and each pair of monomers constitutes a catalytically active dimer. On the other hand, a recent study suggested wheat GS2 of a different cultivar as a hexamer (Wang et al., 2015).

In conclusion we suggest that the fine regulation of wheat GS2 octamer is driven by negative cooperativity. GS2 uses glutamate as allosteric regulator substrate. In this manner the higher the glutamate concentration, the higher the number of working subunit couples within the GS homomer. Thus, it is ensured that GS2 has no possibility of consuming quickly all the available carbon skeletons, but still utilize as much ammonium ion as possible, due to its high affinity to ammonia. This leads to the conclusion that wheat GS2 is not only the key enzyme of the nitrogen metabolism, but itself is an effective balancer between the carbon and nitrogen cycles via its allosteric regulation by negative cooperativity.

## Author contributions

EN, ZN, and AP: Conceived and designed the experiments, performed the measurements; EN: Analyzed the data and wrote the manuscript; EN and AP: Discussed the results.

### Conflict of interest statement

The authors declare that the research was conducted in the absence of any commercial or financial relationships that could be construed as a potential conflict of interest.
