# Corrigendum: Chloroplast Glutamine Synthetase, the Key Regulator of Nitrogen Metabolism in Wheat, Performs Its Role by Fine Regulation of Enzyme Activity via Negative Cooperativity of Its Subunits

**DOI:** 10.3389/fpls.2018.00466

**Published:** 2018-04-24

**Authors:** Edit Németh, Zoltán Nagy, Attila Pécsváradi

**Affiliations:** ^1^Department of Plant Biology, University of Szeged, Szeged, Hungary; ^2^Doctoral School in Biology, Faculty of Science and Informatics, University of Szeged, Szeged, Hungary; ^3^Centre for Agricultural Research, Hungarian Academy of Sciences, Martonvásár, Hungary; ^4^Cereal Research Non-profit Ltd., Szeged, Hungary

**Keywords:** allosteric behavior, glutamine synthetase, negative cooperativity, nitrogen metabolism, *Triticum aestivum*

In the original article, one of the authors, Dr. Dimah Z. Habash, is missing from the authors list from one of the references. The correct reference, which was cited as “(Miflin, 2002)” in the original text, is the following:

Miflin, B. J., and Habash, D. Z. (2002). The role of glutamine synthetase and glutamate dehydrogenase in nitrogen assimilation and possibilities for improvement in the nitrogen utilization of crops. *J. Exp. Bot*. 53, 979–987. doi: 10.1093/jexbot/53.370.979

The authors apologize for the mistake. This error does not change the scientific conclusions of the article in any way.

The original article has been updated.

## Conflict of interest statement

The authors declare that the research was conducted in the absence of any commercial or financial relationships that could be construed as a potential conflict of interest.

