# Supplementary material for: Chloroplast Glutamine Synthetase, the Key Regulator of Nitrogen Metabolism in Wheat, Performs Its Role by Fine Regulation of Enzyme Activity via Negative Cooperativity of Its Subunits
Source: Front Plant Sci. 2018 Feb 19;9:191. doi: 10.3389/fpls.2018.00191 (PMC5827528; doi:10.3389/fpls.2018.00191)
Supplement: Supplementary file 1 [file Image1.pdf]

*Supplementary Material*

# Chloroplast glutamine synthetase, the key regulator of nitrogen metabolism in wheat, performs its role by fine regulation of enzyme activity via negative cooperativity of its subunits

**Edit Németh, Zoltán Nagy, Attila Pécsváradi***

*** Correspondence:** Attila Pécsváradi: pecsvaradi@bio.u-szeged.hu

## 1 Supplementary Figures

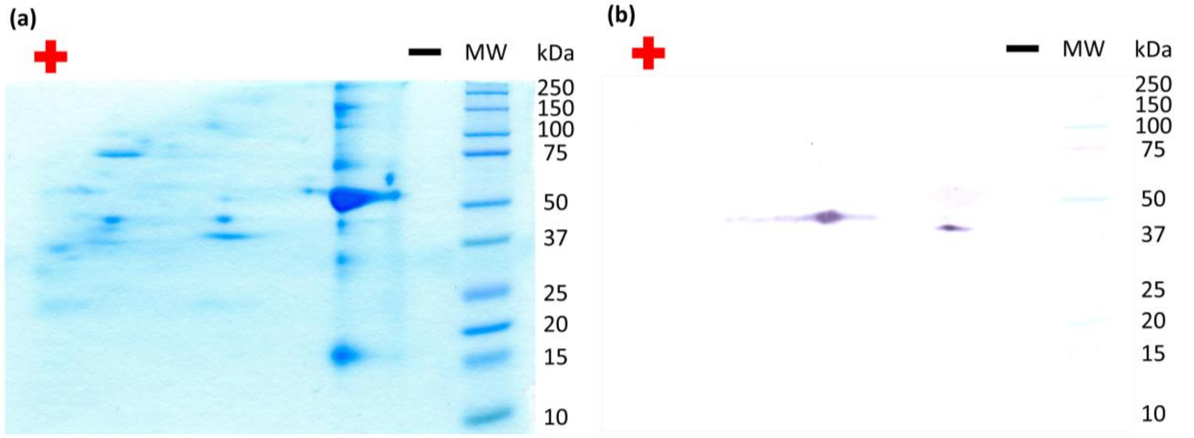


**Supplementary Figure 1.** Original gel and blot presented on figure 1. (a) Coomassie Brilliant Blue G stained 2D PAGE separation of crude leaf extract; (b) Immunoblot of glutamine synthetase after 2D PAGE separation showing the different localization of GS isoenzymes in polyacrylamide gel. Plus and minus labels the anode and cathode site of the first dimension respectively. MW: molecular weight standard.
